# Passive Filler-Loaded Silicon Oxycarbide Coating on Nickle Alloy with High Thermal Shocking Behavior and Oxidation Resistance

**DOI:** 10.3390/ma15186395

**Published:** 2022-09-15

**Authors:** Zhengkai Tian, Wenxia Zhu, Xiao Yan, Dong Su

**Affiliations:** 1 Key Laboratory of Advanced Ceramics and Machining Technology of Ministry of Education, School of Materials Science and Engineering, Tianjin University, Tianjin 300350, China; 2 Shenzhen Institute of Information Technology, Shenzhen 518172, China

**Keywords:** SiOC coating, thermal protection, passive filler, crack mechanism

## Abstract

Polymer-derived ceramic (PDC) coatings of considerable thickness can offer promising protection for metallic and superalloy substrates against oxidation and corrosion, yet the preparation remains challenging. Here, a SiOC/Al_2_O_3_/YSZ coating was prepared on a nickel alloy with a spraying method using Al_2_O_3_ and yttria-stabilized zirconia (YSZ) as passive fillers. The thickness can reach up to 97 μm with the optimal mass fraction and particle sizes of the passive fillers. A small or isolated SiOC phase is formed in the coating, which can effectively alleviate the shrinkage and cracking during the pyrolysis. The SiOC/Al_2_O_3_/YSZ coating exhibits low thermal conductivity and high bonding strength with the substrate. Moreover, the coating shows good thermal shock resistance between 800 °C-room temperature cycles and oxidation resistance at 1000 °C for 36 h. This work provides an effective guide for the design of thick PDC coatings to further promote their application in the thermal protective field.

## 1. Introduction

High-temperature protective coatings are necessary for metallic and superalloy substrates that serve in highly reactive and corrosive environments (such as oxidation and acidic/alkalic corrosion), where the unprotected metals degrade quickly or even fail. The coatings are designed to extend the service time of the substrates and thus enhance the safety and reduce the costs during their usage at high temperature [1,2]. Ceramic-based coatings with considerable thickness are urgently needed for thermal protective systems for their high service temperature, long service time, and high reliability under harsh environments [3]. Especially, the development of high-performance ceramic-based coatings for superalloy parts in the hottest part of gas-turbine engines and jet engines is promising, as they will be directly exposed to high-low temperature cycling and oxidizing environments in thermal management systems, aiming to further improve the overall performance (e.g., increased energy efficiency, thrust-to-weight ratio, and durability) [4]. Therefore, novel materials with desired structure and properties are needed.

Silicon-based precursor-derived ceramics (PDCs) exhibit excellent thermal stability, creep, corrosion, and oxidation resistance at high temperatures [3], which makes them promising candidates as high-temperature resistant materials for structural applications in harsh environments. Moreover, PDCs can be feasibly processed through various techniques for developing low-dimensional ceramic materials such as fibers and coatings [5,6]. SiOC [7,8,9,10] and SiCN(O) [11,12,13] coatings have been explored on steel and nickel-based alloys by various deposition techniques such as dipping [7,8,9,13] and spinning [14,15,16] techniques to provide oxidation and corrosion resistance. Dip coating and spin coating are the most commonly used methods for the preparation of PDC coatings, mainly because the required equipment is relatively simple and the process is reproducible. However, the edge effect greatly affects the thickness homogeneity of dip coating, especially on the substrates with small sizes or complex shapes. The spinning method is mostly suitable for flat or slightly curved surfaces and is mostly required for liquids with low viscosity. In addition, the thicknesses of spinning coatings are usually small in the range of a few nanometers to a few micrometers. Recently, the spraying method was also used to prepare a SiCN coating [15,16]. Compared with previous methods, spray depositing makes it easy to control the characteristics of coatings (e.g., thickness and surface structure) by adjusting the preparation parameters. Moreover, the spray method also supports parts with large sizes and complex shapes for the industrial requirement. So, the spraying method is a promising route to prepare PDC coatings. The PDC coatings have also exhibited promising potential applications in energy, environment, and transportation [6,17]. Unfortunately, during the organic-to-inorganic transition of the polymer precursors, stresses arise in the PDC network due to the gas evolution and large shrinkage (up to 70 vol.%), leading to the cracking of PDC coatings [18,19,20]. Moreover, thermal expansion mismatch between PDCs and metallic substrates also contribute to the cracking of PDC coatings due to the low coefficient of thermal expansion (CTE) [21]. This is especially the case in the preparation of thick coatings, where the mismatch often leads to the degradation or failure of the PDC coatings, severely hindering their practical usage. Up to now, the pure PDC coatings that are applicable on metallic substrates could merely have small thicknesses of lower than 2 μm [11].

Passive fillers (such as ZrO_2_ [22,23,24] and Al_2_O_3_ [25]) or active fillers (such as Al [25], TiSi_2_ [7,8,9,10,19], and ZrSi_2_ [13]) can be introduced into PDC coatings to alleviate the shrinkage of precursors during the polymer-to-ceramic transition, facilitating the preparation of thicker PDC coatings (generally 15~50 μm) [7,8,9,10,13]. Moreover, the fillers can further enhance the properties of PDC coatings [11,26,27]. For example, yttria-stabilized zirconia- (YSZ-) filled SiCN coating showed low thermal conductivity [28] and a high coefficient of thermal expansion [29]. However, the introduction of fillers increases the porosity of the PDC coatings; moreover, despite the reduction of the macroscopic shrinkage, crack formation may still occur due to localized stresses (e.g., around filler particles), especially in thicker PDC coatings [23,24,25]. For instance, meltable glass fillers (such as Glass G8470 and Glass G018-311) [22,23,24,25,30] in PDC coating could help facilitate the release of internal stress and the formation of liquid at high temperature, which can seal the cracks generated in the coating, resulting in an enhanced thickness of the SiCN coating (over 90 μm [23,24]). However, the liquid glass phase with a high viscosity could hinder the escaping of gas molecules during the pyrolysis of precursors, further forming large-sized defects such as cracks and pores in the PDC coating [23,24,25]. Moreover, the existence of a low-melting glass phase in PDC coating limited the service temperature of the coating (lower than 750 °C [23,24,25]).

Therefore, up to now, it has been a major challenge to obtain thick, crack-free PDC coatings on metallic/superalloy substrates. The thinness of the PDC-based coatings severely limits their thermal barrier behavior for metallic substrates and the protective abilities of oxidation and corrosion, hindering their practical usage in high-temperature protection. Moreover, as far as our knowledge is concerned, most previous works focused on the oxidation or corrosion resistance performance of PDC coatings. Meanwhile, the thermal shock stability and bonding strength of the coatings were rarely explored, probably due to the defects in the coating, which also severely restricts their practical usage in harsh environments.

In this paper, a SiOC/Al_2_O_3_/YSZ coating was prepared on the K3 nickel alloy by spraying method using Al_2_O_3_ and yttria-stabilized zirconia (YSZ) as passive fillers, in which the YSZ filler was used to enhance the thermal expansion coefficient of the coating to match the K3 substrate, and the Al_2_O_3_ filler was used to further adjust the thermal matching between the YSZ filler and SiOC ceramic. The cracking mechanism of passive-loaded SiOC coating was explored in detail by changing the mass fraction and the particle size of the passive fillers in the coating system to provide an effective guide for the preparation of crack-free thick coatings. The thickness of the SiOC/Al_2_O_3_/YSZ coating can be up to 97 μm. Moreover, the SiOC/Al_2_O_3_/YSZ coating shows good mechanical and thermal properties, including high bonding strength with the substrate, low thermal conductivity, and good high-low temperature cycling stability and oxidation resistance. This work could further promote the development and application of PDC as thermal protective coatings in harsh environments.

## 2. Experimental Section

### 2.1. Materials

Commercially available polyhydromethylsiloxane (PHMS, Me_3_SiO[MeHSi-O]_n_SiMe_3_, Shenzhen Jipeng Silicon Fluoride Materials, China) and tetramethyl-tetravinylcycletetrasiloxane (D_4_Vi, [MeViSiO]_4_, Shenzhen Jipeng Silicon Fluoride Materials, Shenzhen, China) were used as polysiloxane (PSO) precursor for the SiOC coating, and platinum complex ([Pt]: 3000 ppm, Shenzhen Hongye Silicone Materials, ShenZhen, China) was used as a catalyst for the PSO precursor. The 8 wt.% yttria-stabilized zirconia powder (YSZ, average particle size: 50 nm and 1 μm, Xingtai Xindun Metal Materials Co., Ltd. Hebei, China) and Al_2_O_3_ powder (average particle size: 5 μm, Shanghai Meryer Chemical Technology Co. Ltd., Shanghai, China) were used as passive fillers for the SiOC coating, and ethanol (purity > 99.7%, Tianjin Yuanli Chemical Co. Ltd., Tianjin, China) was used as the solvent. K3 nickel alloy sheets (10 mm × 10 mm × 2 mm, Beijing Institute of Aeronautical Materials, Beijing, China, long-serving temperature ≤ 1000 °C), which were used as the substrates, were polished with 400 mesh silicon carbide sandpaper and then cleaned in ethanol under ultrasonic treatment, followed by drying at ambient conditions.

### 2.2. Preparation of SiOC/Al_2_O_3_/YSZ Coating

The SiOC/Al_2_O_3_/YSZ coating was prepared on the K3 substrate through slurry spraying, crosslinking, and pyrolysis. As is the standard process, 1.25 g of PHMS, 1.25 g of D_4_Vi, 4.5 g of Al_2_O_3_, 4.5 g of YSZ, and 26.5 g of ethanol were mixed under a ball mill for 2 h to obtain a uniform PSO/Al_2_O_3_/YSZ suspension. Then, 0.17 g of Pt catalyst was added while stirring for 30 min to form a PSO/Al_2_O_3_/YSZ slurry. Subsequently, the slurry was sprayed on the K3 substrates using a spray gun (Shanghai Tewei Spraying Technology Co., Ltd. Shanghai, China) with pressurized air as the carrier gas. The spraying parameters were as follows: nozzle diameter of 1.0 mm, air pressure of 0.4–0.7 MPa, spraying distance of 10 cm, and spraying speed of 10 cm/s. Afterward, the PSO/Al_2_O_3_/YSZ coatings were dried and crosslinked at 60 °C for 120 min. Finally, the coatings were pyrolyzed in a tube furnace in argon at 1000 °C for 2 h with a heating rate of 3 °C/min to form SiOC/Al_2_O_3_/YSZ coatings.

For comparison, a serial of the SiOC/Al_2_O_3_/YSZ coatings were obtained by changing the mass fraction of the fillers of Al_2_O_3_ and YSZ in the coating system (68 wt.%, 75 wt.%, and 79 wt.%), the mass ratio of Al_2_O_3_/YSZ (0:1, 2:3, 1:1, 3:2 and 7:3), the particle size of YSZ (average particle size: 50 nm and 1 μm), and the number of spraying (30~90 times).

### 2.3. Characterization

The morphology and elemental distribution of the SiOC/Al_2_O_3_/YSZ coatings were characterized by field emission scanning electron microscopy (SEM, TDCLS-S4800, Hitachi, Japan) equipped with an energy-dispersive spectrometer (EDS). The thickness of the coating was calculated by the cross-sectional SEM image, and the edge portions of the coating on the Ni substrate were chosen for characterization. The crystalline structure was identified by X-ray diffraction (XRD, D/Max–2500, Rigaku, Tokyo, Japan) using CuK_α_ radiation (λ = 1.5418 Å) in the range of 10°–80° with a step size of 0.02°/step and a step time of 0.12 s/step in fixed scan mode. The interface bonding strength between the coating and the substrate was measured by a pull-off method on the samples (1 cm × 1 cm × 2 mm) using an integrated mechanical testing machine (XWW, Jinshengxin, Beijing, China) with a stretching speed of 0.5 mm/min. Thermal conductivity at room temperature was measured by a hot wire method (TC3000, XIATECH, Xian, China) on a cylindrical sample (Φ 5 cm × 2 mm) with a similar composition of the optimal coating. Thermal conductivity at high temperature was measured by a laser method (LFA457, Netzsch, Selb, Germany) in the range of 25–1000 °C on a cylindrical sample (Φ 12.7 mm × 2 mm) prepared by thermal dry-pressing. The SiOC/Al_2_O_3_/YSZ sample had a similar composition to the optimal coating but high density (1.9 g/cm^3^), and the SiOC sample had a density of 1.8 g/cm^3^. The thermal shocking property was exposed under 800 °C for 1 h in an air furnace (SX-5-12, Yixing Qianjin furnace equipment Co., Ltd., Yixing, China) and then quickly cooled to room temperature in water (~25 °C). Thermal shock cycling was performed until the coating cracked. The oxidation test was carried out in an air furnace (SX-5-12, Yixing Qianjin furnace equipment Co., Ltd., Yixing, China) at 1000 °C for 12 h, 24 h, and 36 h.

## 3. Results and Discussion

### 3.1. Preparation and Structure Regulation

**Figure 1:**
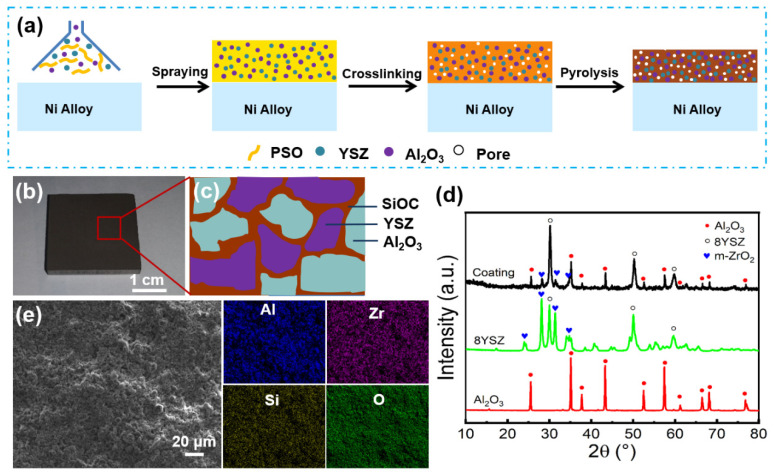
(**a**) Schematic illustration of preparation process of the SiOC/Al_2_O_3_/YSZ coatings, (**b**) photograph, (**c**) simulation of phase structure, (**d**) XRD patterns, (**e**) SEM image and elemental mappings of the SiOC/Al_2_O_3_/YSZ coating: blue for Al, purple for Zr, yellow for Si, and green for O.

The SiOC/Al_2_O_3_/YSZ coating was prepared on K3 alloy substrates by the slurry spraying method followed by crosslinking and pyrolysis, as shown in Figure 1a. The PSO/Al_2_O_3_/YSZ slurry was continuously deposited on the K3 substrates, resulting in the increased thickness of the coating after the evaporation of ethanol solvent. The organic PSO precursor in the coating was further crosslinked by the hydrosilylation reaction of PHMS and D_4_Vi during the drying then transformed into an amorphous rigid SiOC network after pyrolysis [31]. Figure 1b shows a uniform and crack-free SiOC/Al_2_O_3_/YSZ coating with a size of 2.5 cm × 2.5 cm applied on the K3 substrate. On the one hand, the SiOC phase as ceramic binder distributes among the Al_2_O_3_ and YSZ fillers to connect them to form an intact coating (Figure 1c). On the other hand, the SiOC phase exhibits good wettability and cohesiveness with the K3 substrate, which endow the coating with a high bonding strength with the substrate.

The XRD pattern of the SiOC/Al_2_O_3_/YSZ coating shows strong characteristic peaks of the Al_2_O_3_ crystalline phase (JCPDS no.9-0036, 2θ = 35.2°, 43.4°, 57.5° and 68.2°) and 8YSZ crystalline phase ((Zr_0.94_Y_0.06_)O_1.88_, JCPDS no.89-9068, 2θ = 30.2°, 50.2°, 50.6°, and 60.1°), as well as small diffraction peaks for the *m*-ZrO_2_ crystalline phase (JCPDS no.86-1450, 2θ = 24.1°, 28.2°, 31.5°, and 34.2°), corresponding to the Al_2_O_3_ and YSZ fillers in the coating (Figure 1d). The EDS elemental mappings show that the elements of Al, Zr, Si, and O were homogenously distributed on the surface of the coating (Figure 1e).

**Figure 2:**
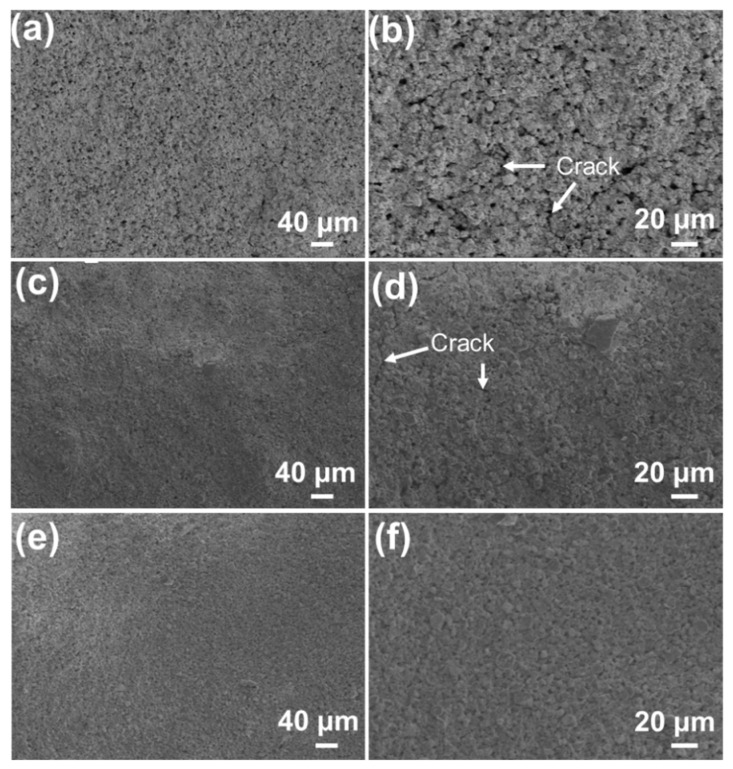
SEM images of the surface sections of the SiOC/Al_2_O_3_/YSZ coatings with different mass fractions of Al_2_O_3_ and YSZ: (**a**,**b**) 68 wt.%, (**c**,**d**) 75 wt.%, (**e**,**f**) 79 wt.%.

The SiOC/Al_2_O_3_/YSZ coatings were prepared by changing the mass fraction of the Al_2_O_3_ and YSZ fillers in the system (68 wt.%, 75 wt.%, and 79 wt.%). All coatings are well-combined with the K3 substrate without obvious macroscopic cracking, which is quite different from the pure SiOC coating, showing the alleviation effect of the introduction of Al_2_O_3_ and YSZ fillers on the volume shrinkage of the SiOC during the pyrolysis. However, under high-resolution SEM observation, some defects (e.g., pores and microcracks) inevitably appeared on the surface of the low-filler-loaded SiOC/Al_2_O_3_/YSZ coating, as shown in Figure 2a,b. The size and amount of the cracks decreased with the increased fillers’ fraction (Figure 2c,d). When the fraction of the fillers was increased to 79 wt.%, the cracks completely disappeared on the surface (Figure 2e,f), indicating that the stress caused by the SiOC shrinkage during the pyrolysis could be effectively reduced to the lowest level by adding enough fillers in the coating, which is similar to the filler-loaded bulky PDCs [20]. However, further increasing the fillers’ fraction would lead to a serious decrease in the strength of the SiOC/Al_2_O_3_/YSZ coating due to insufficient adhesion of the SiOC phase to the Al_2_O_3_ and YSZ fillers.

**Figure 3:**
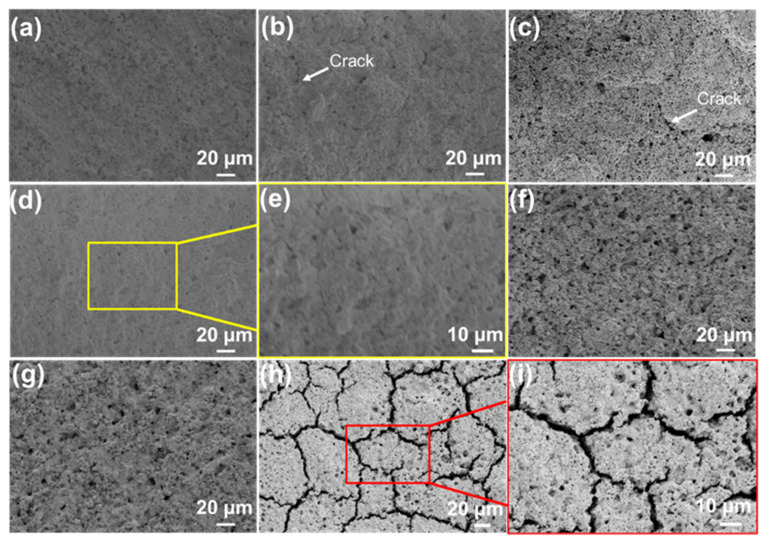
SEM images of the surface sections of the SiOC/Al_2_O_3_/YSZ coatings using YSZ filler (particle size 1 μm) by changing the mass ratio of Al_2_O_3_ and YSZ: (**a**,**b**) 0:1, (**c**) 2:3, (**d**,**e**) 1:1, (**f**) 3:2, (**g**) 7:3; and (**h**,**i**) using YSZ filler (particle size 50 nm) under the mass ratio of Al_2_O_3_ and YSZ of 1:1. Spraying number: (**a**) 10 times, (**b**) 30 times, others 50 times.

The decreased flaws in the SiOC/Al_2_O_3_/YSZ coatings with increased fractions of fillers indicate the effective contribution of fillers to the inhibiting the volume shrinkage of SiOC during the pyrolysis. The mass ratio of Al_2_O_3_ and YSZ in the system was further changed to 0:1, 2:3, 1:1., 3:2, and 3:7 to prepare the coating. When only the YSZ filler was used, the thinner SiOC/YSZ coating, obtained by spraying 10 times, was intact and homogenous (Figure 3a). However, with an increase in spraying times to 30 (Figure 3b), the coating gradually cracks, or after spraying 50 times, it even peels off from the K3 substrate. The crack in the SiOC/YSZ coating was mainly due to the thermal expansion mismatch between SiOC and YSZ that led to stress accumulation in the coating. When the Al_2_O_3_ filler was added to the coating, it was easy to prepare thicker coatings by spraying 50 times or more (Figure 3c). Increasing the mass ratio of Al_2_O_3_/YSZ to 1:1, the SiOC/Al_2_O_3_/YSZ coating became uniform and crack-free (Figure 3d,e). However, further increasing the mass ratio of Al_2_O/YSZ to 3:2 and 7:3, the SiOC/Al_2_O_3_/YSZ coatings became rough and formed some macropores on their surfaces with sizes of 2–5 μm (Figure 3f,g). These pores were formed by the packing of filler particles due to insufficient adhesion of the SiOC phase to the Al_2_O_3_ and YSZ fillers, further resulting in reduced strength of the coating. Therefore, the optimal mass ratio of Al_2_O_3_ and YSZ fraction in the coating should be 1:1.

Moreover, the particle size of the passive filler affects the preparation of the SiOC/Al_2_O_3_/YSZ coating. When the particle size of the YSZ filler decreased to 50 nm, high amounts of connected cracks with large scale (width: 2 μm) were observed on the surface of the coating (Figure 3h,i), indicating that nanoparticles are too small to hinder the volume shrinkage of SiOC during the pyrolysis. Therefore, the micron-size filler should be suitable to obtain a thick SiOC-based coating.

**Figure 4:**
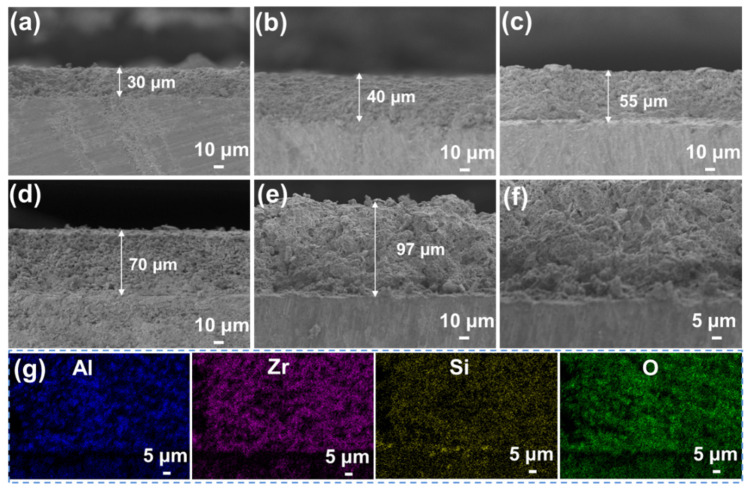
SEM images of the cross-sections of the SiOC/Al_2_O_3_/YSZ coatings with different spraying times: (**a**) 30 times, (**b**) 40 times, (**c**) 50 times, and (**d**) 60 times; (**e**) the thickest coating obtained and (**f**) its enlarged SEM images of interface between the coating and the substrate, (**g**) elemental mappings of the cross-section of the SiOC/Al_2_O_3_/YSZ coating, blue for Al, purple for Zr, yellow for Si and green for O.

Under optimal conditions, the thick SiOC/Al_2_O_3_/YSZ coatings are successfully prepared by increasing the spraying times. As shown in Figure 4a–d, the thickness of the coating increased from 30 μm to 70 μm controlled by the increased spraying from 30 times to 60 times. In general, the thickness of the coating increases by 10–15 μm for each 10 sprays. The maximum thickness of the crack-free SiOC/Al_2_O_3_/YSZ coating is up to 97 μm with the roughness of ~1 μm (Figure 4e), which is much higher than the previous passive filler-loaded PDC coatings [13,15,16,28] (<50 μm), and close to the maximum thickness of the glass-filler-loaded PDC coatings (up to 93 μm), as shown in Table 1. Though the glass-filler-loaded PDC coating had a greater thickness, many defects, such as cracks and large pores, could be easily observed [23]. Moreover, the presence of low-melting glass in the coating limited the usage temperature of the coating (usually lower than 750 °C) [23].

The enlarged SEM image (Figure 4f) shows that the thick coating is closely bonded with the K3 substrate with a clear interface between the coating and the substrate. The EDS elemental mappings show that the elements of Al, Zr, Si, and O are homogenously distributed on the cross-section of the coating (Figure 4g). The increased thickness of the SiOC/Al_2_O_3_/YSZ coating could ensure its structural strength and high-temperature resistance during usage. The average interfacial bonding strength of the SiOC/Al_2_O_3_/YSZ coating was 16.6 MPa with the highest value of 21.0 MPa, indicating the good combination of the coating with the K3 substrate coming from the good wettability and cohesiveness of the SiOC phase with the substrate.

### 3.2. Model Derivation

**Figure 5:**
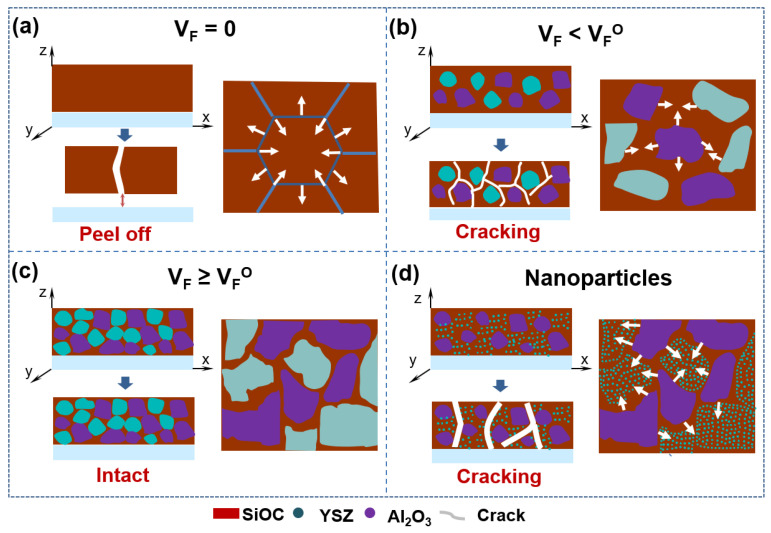
Schematic model of cracking mechanism in the SiOC/Al_2_O_3_/YSZ coating system: (**a**) cracking mechanism of PSO-free coatings, (**b**) cracking mechanism of coatings with PSO less than critical value, (**c**) cracking mechanism of coatings with PSO greater than or equal to critical value, (**d**) cracking mechanism of coatings with nanoscale filler particles. Blue arrowhead: transformation of coatings before and after heat treatment, white arrowhead: shrinkage trend of coatings.

The transition of organic PSO precursor to inorganic SiOC ceramic is a complex physical and chemical process accompanied by the cleavage of C-H and rearrangement of Si-O and Si-C bonds during pyrolysis, leading to the evolution of the amount of the hydrocarbon gases, resulting in large mass loss (20–30 wt.%) [32] as well as large volume shrinkage, creating large internal stress inside the SiOC system [33]. Moreover, in the case of coating, the shrinkage is constrained by the adhesion of the coating to the substrate, leading to larger tensile stresses, which increase the likelihood of cracks in the coating. This is particularly the case in the fabrication of PDC coating with greater thickness. The magnitude of the internal stress in the range of 10^−2^–10^3^ MPa is positively related to the shrinkage rate of the precursors during pyrolysis [34]. For pure SiOC coating, the bonding strength between the coating and the substrate is far lower than the internal stress, initiating cracks in the coating and eventually peeling off from the substrate, as shown in Figure 5a.

It is well known that fillers in the SiOC system can effectively relieve shrinkage to reduce the internal stress during pyrolysis, thereby avoiding the occurrence of cracking [20]. For the PDCs/filler system, the volumetric shrinkage of the system (w_v_) after pyrolysis could be estimated by Equation (1) [20]. The volume shrinkage of the PDCs/filler system is negatively correlated with the volume fraction of the filler. When the volume fraction of the filler reaches $V_{F}^{*}$, the volume shrinkage of the system is zero. According to the packing theory, we can obtain the maximum packing rate of 74 vol.% (i.e., $V_{F}^{*}$) when the equal-diameter spheres are tightly packed.

(1) $$w_{V}=\left(1-\frac{V_{F}}{V_{F}^{*}}\right)w_{V}\left(P\right)$$

where V_F_ is the volume fraction of the inert filler, $V_{F}^{*}$ is the critical volume fraction of the inert filler (maximum packing density) and w_v_(P) is the volumetric shrinkage of the preceramic polymer.

As for the coating system, the crack in the SiOC coating can also be mainly attributed to the fillers’ fraction of the bulky materials. According to our experiment, the optimal volume fraction of the fillers (donated as $V_{F}^{O}$) in the SiOC/Al_2_O_3_/YSZ coating is ~52 vol.%, which is less than $V_{F}^{*}$ of 74 vol.% in the bulk. The SiOC phase in the coating mainly distributes in the area among the fillers, and its scale and distribution state are closely related to the fillers’ fraction in the coating, further affecting the inhibitory effect on the SiOC shrinkage. The smaller and thinner the formed SiOC phase, the lower the shrinkage stress during the pyrolysis, which could effectively suppress the cracking of the coating. When 0 < V_F_ < $V_{F}^{O}$ (Figure 5b), the SiOC phase is wholly connected with a large size, and then large stress generated by the shrinkage of the SiOC tends to result in cracking in the coating. When V_F_ ≥ $V_{F}^{O}$ (Figure 5c), the SiOC phase gradually tends to form isolated islands in the coating or thin layers on the surface of the fillers, and then the stress is too small to form a crack in the coating. In addition, as shown in Figure 5d, the nano-fillers tend to distribute in the SiOC phase, and the large size of the SiOC phase would increase the thermal stress caused by the volume shrinkage during the pyrolysis. Therefore, the addition of nano-sized fillers into the SiOC/Al_2_O_3_/YSZ coating shows a weak inhibitory effect on the cracking (Figure 3h,i).

### 3.3. Thermal Conductivity Test

The thermal conductivity of the best SiOC/Al_2_O_3_/YSZ coating system was investigated by hot wire method, resulting in an outstanding value of 0.62 ± 0.02 W m^−1^∙K^−1^, which was lower than that of typical values (1.0–1.5 W m^−1^∙K^−1^) of the YSZ thermal barrier coatings prepared by plasma spraying and electron beam physical vapor deposition [35,36].

The thermal conductivity of the SiOC/Al_2_O_3_/YSZ coating at high temperatures was further measured by laser method. The temperature-dependent thermal conductivity *λ(T)* can be calculated according to Equation (2):

(2) $$\lambda \left(T\right)=\alpha \left(T\right)\cdot C_{p}\left(T\right)\cdot \rho \left(T\right)$$

where *α(T)* is the temperature-dependent thermal diffusivity, *C_p_(T)* is the temperature-dependent specific heat capacity, and *ρ(T)* is the temperature-dependent density.

**Figure 6:**
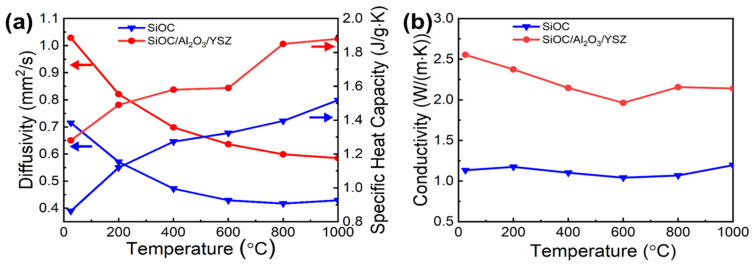
Thermal properties of the SiOC ceramic and the SiOC/Al_2_O_3_/YSZ ceramic as a function of temperature: (**a**) thermal diffusivity and specific heat capacity, (**b**) thermal conductivity.

The SiOC ceramic prepared by dry pressing showed a low thermal conductivity of ~1.2 W m^−1^∙K^−1^ during the range of 25–1000 °C, indicating it is a suitable matrix for thermal protection. For the SiOC/Al_2_O_3_/YSZ ceramic (Figure 6a,b), the thermal conductivity decreased gradually with the increase of temperature between 0–600 °C due to the gradual decrease of the thermal diffusivity of conduction and heat transfer; however, it slightly increased with temperature between 600 °C–1000 °C due to the gradual increase of the specific heat capacity of radiative heat transfer. The thermal conductivity of the SiOC/Al_2_O_3_/YSZ ceramic is 2.1 W m^−1^∙K^−1^ at 1000 °C, which is slightly lower than the ZrO_2_ thermal barrier coating of 2.2 W m^−1^∙K^−1^ [28], implying its good thermal insulation property and promising candidacy as a thermal barrier coating.

### 3.4. Thermal Shock Test

**Figure 7:**
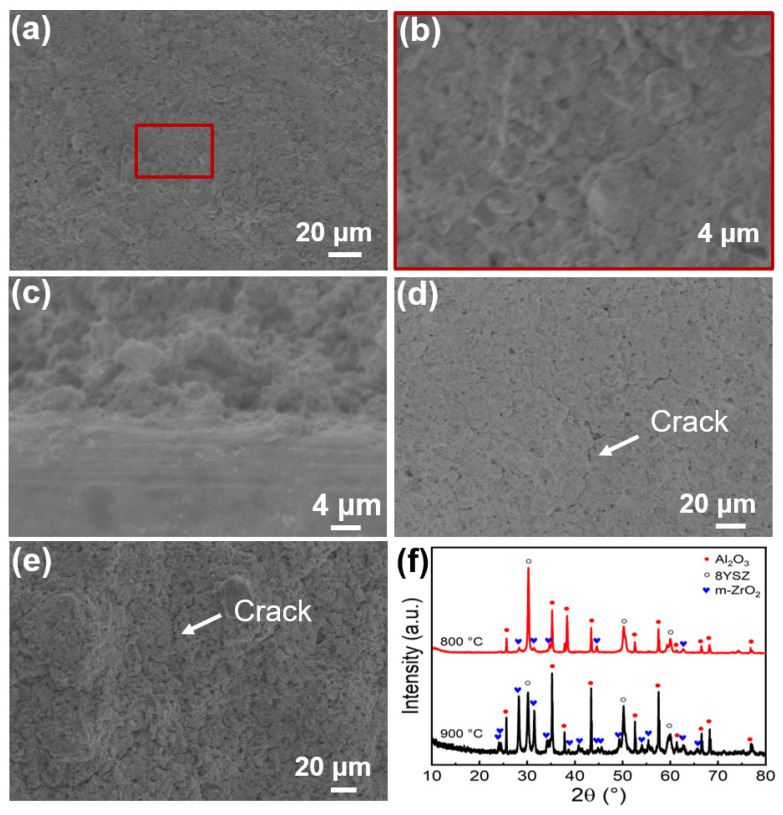
SEM images of the SiOC/Al_2_O_3_/YSZ coatings after thermal shock test: (**a**–**c**) after 6 cycles during 800 °C-RT: (**a**) and (**b**) surface-section, (**c**) cross-section; (**d**) after 7 cycles during 800 °C-RT; (**e**) after 1 cycle during 900 °C-RT; and (**f**) XRD patterns of SiOC/Al_2_O_3_/YSZ coatings after thermal shock test during 800 °C-RT and 900 °C-RT after 6 cycles.

The thermal shocking property of the SiOC/Al_2_O_3_/YSZ coating was explored under high temperature (at 800 °C in the furnace)—low temperature (at room temperature (RT) in water) cycling to verify its feasibility for thermal protection application in extreme environments. After six cycles, the SiOC/Al_2_O_3_/YSZ coating remained intact and crack-free (Figure 7a,b), and was well bonded to the K3 substrate (Figure 7c). After seven cycles, the coating was still intact and flat without any macroscopic crack. Yet, under high-resolution SEM observation, a few micro-cracks appeared on its surface (Figure 7d) caused by the continuous accumulation of thermal stress inside the coating during the cycling. After further increasing the test temperature to 900 °C, microcracks appeared on the surface of the SiOC/Al_2_O_3_/YSZ coating (Figure 7e). As far as our knowledge is concerned, the research on the thermal shock behavior of PDC coatings is limited, and our SiOC/Al_2_O_3_/YSZ coating exhibits excellent stability under thermal cycling.

The phase composition of the SiOC/Al_2_O_3_/YSZ coating after the thermal shock test was further analyzed by XRD. Compared with the original coating (Figure 1d), no change in the phase compositions (mainly Al_2_O_3_ and 8YSZ, a small number of *m*-ZrO_2_) was observed on the coating after six thermal shock cycles during 800 °C-RT (Figure 7f), indicating the phase stability during the cycling. After 900 °C-RT cycling, the *m*-ZrO_2_ crystalline phase in the coating increased in response to the stress-induced transformation of *t*-ZrO_2_ phase to *m*-ZrO_2_ phase during the cycling, which may have led to the volume expansion and then forming microcracks inside the coating (Figure 7e).

### 3.5. Oxidation Resistance Test

**Figure 8:**
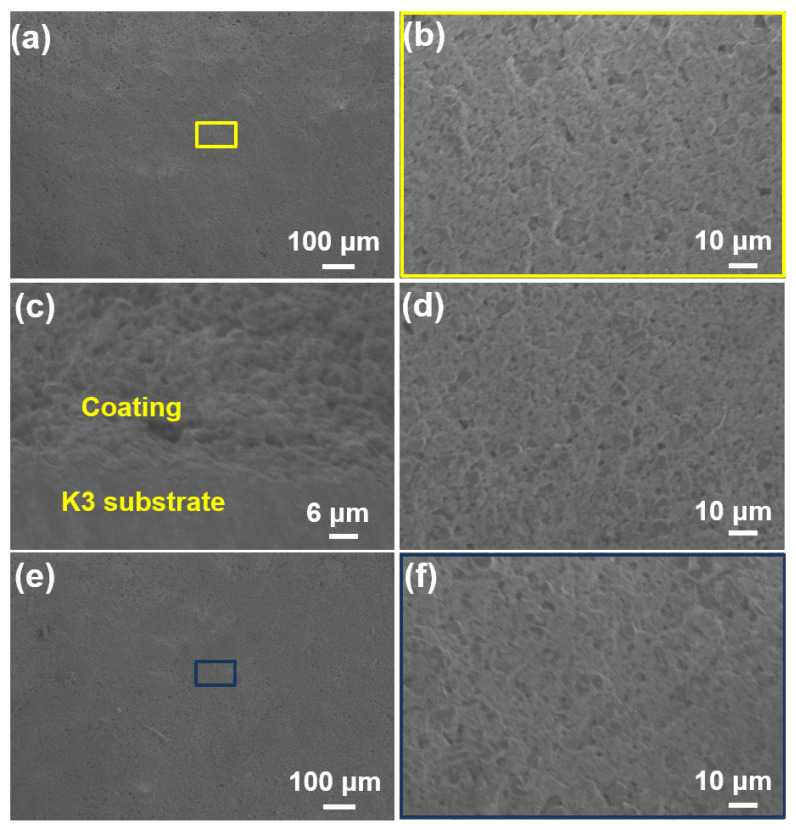
SEM images of the SiOC/Al_2_O_3_/YSZ coatings after oxidation test at 1000 °C for different times: (**a**–**c**) for 12 h, (**a**,**b**) surface-section, (**c**) cross-section; (**d**) for 24 h, surface-section; and (**e**,**f**) for 36 h, surface-section.

The oxidation resistance test on the SiOC/Al_2_O_3_/YSZ coating was carried out at 1000 °C in a static air environment over time periods of 12 h, 24 h, and 36 h. After 12 h oxidation, the surface of the coating remained flat, uniform, and crack-free (Figure 8a,b) as before oxidation, and the cross-section showed close bonding between the coating and the substrate without any cracks (Figure 8c,d). Even after 24 h and 36 h oxidation, the SiOC/Al_2_O_3_/YSZ coating was still intact and crack-free **(**Figure 8e,f), further indicating the excellent oxidation of the coating at 1000 °C in a static environment.

**Figure 9:**
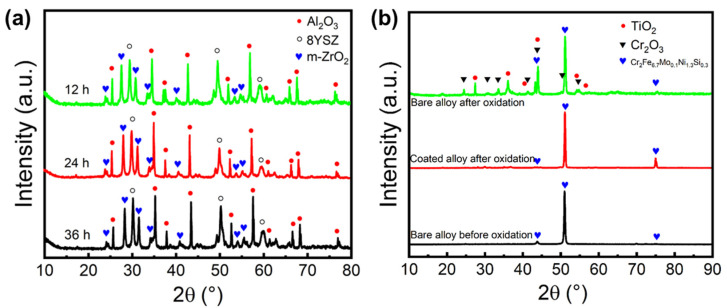
XRD patterns of (**a**) the SiOC/Al_2_O_3_/YSZ coatings after oxidation test at 1000 °C for 12 h, 24 h, and 36 h, and (**b**) the K3 alloy substrate with and without the coating after oxidation test at 1000 °C for 36 h.

The phase composition of the SiOC/Al_2_O_3_/YSZ coating after the oxidation resistance test was further analyzed by XRD. Compared with the original coating (Figure 1d), no change in the phase compositions (mainly Al_2_O_3_, 8YSZ, and m-ZrO_2_) was observed on the coating after oxidation (Figure 9a), and the slightly increased m-ZrO_2_ was mainly due to the stress-induced phase transition of t-ZrO_2_. Interestingly, there was no change in the t-ZrO_2_ phase and m-ZrO_2_ phase of the coatings after increased oxidation time (12 h, 24 h, and 36 h), as shown in Figure 9a, further indicating the excellent oxidation resistance of the SiOC/Al_2_O_3_/YSZ coating. The XRD pattern of the K3 alloy substrate without coating before oxidation shows strong characteristic peaks of the Cr_2_Fe_6.7_Mo_0.1_Ni_1.3_Si_0.3_ crystalline phase (JCPDS no.50-1292, 2θ = 43.9°, 51.0°, 75.4°, Figure 9b). After 36 h oxidation at 1000 °C, the K3 substrate without coating shows two other strong characteristic peaks of the Cr_2_O_3_ crystalline phase (JCPDS no.38-1479, 2θ = 24.5°, 33.6°, 36.2°, 54.9°) and TiO_2_ crystalline phase (JCPDS no.21-1276, 2θ = 27.4°, 36.1°, 41.2°, 54.3), indicating the poor oxidation resistance of the K3 substrate. Differently, the K3 substrate with the coating only shows strong characteristic peaks of the Cr_2_Fe_6_._7_Mo_0_._1_Ni_1_._3_Si_0_._3_ crystalline phase but without the appearance of oxides after 36 h oxidation. So, it is clearly shown that various oxides (such as Cr_2_O_3_ and TiO_2_) appeared on the Ni substrate without coating due to high temperature oxidation, while no oxide was observed on the Ni substrate with the SiOC/Al_2_O_3_/YSZ coating, indicating the effective anti-oxidation protection of the K3 substrate by the SiOC/Al_2_O_3_/YSZ coating.

Therefore, low thermal conductivity, high bonding strength, outstanding oxidation resistance, and thermal shocking behavior have been simultaneously achieved on our SiOC/Al_2_O_3_/YSZ coating, making it an ideal candidate for thermal protection applications in harsh environments.

## 4. Conclusions

A SiOC/Al_2_O_3_/YSZ coating was successfully prepared on a K3 nickel alloy substrate by the slurry spraying method followed by crosslinking and pyrolysis. The thickness of up to nearly 100 μm was successfully obtained under the optimal conditions (mass fraction of fillers of 79 wt.%, mass ratio of Al_2_O_3_/YSZ of 1:1). The low fraction or small particle sizes of passive fillers in the coating system could lead to the cracking or peeling off of the coatings due to the large stress during the pyrolysis, and the mismatching of thermal expansion between the fillers and the SiOC could generate microcracks in the coatings. The SiOC/Al_2_O_3_/YSZ coating exhibits low thermal conductivity and high bonding strength with the K3 substrate. Moreover, it shows good thermal shock resistance and oxidation resistance. This work provides an effective guide for the design and preparation of thicker PDC coatings to expand their application in the thermal protection field.

## Figures and Tables

**Table 1:** Comparison of PDC-based coatings.

PDCs	Passive or Active Fillers	Glass Fillers	Deposition Method	Thickness (μm)	Oxidation Test (°C/h)	Refs.
SiCN	Si_3_N_4_	-	Dipping	1.5	1000/10	[11]
SiOC	TiSi_2_	-	Dipping	18.0	-	[7,8,9]
SiOC	TiSi_2_	-	Dipping	20.0	800/100	[10]
SiOC	ZrSi_2_	-	Dipping	25.3	800/50	[13]
SiCN	ZrO_2_	Glass G8470, Glass G018-311	Spraying	20.0	-	[15]
SiCN	Al/ZrO_2_	-	Spraying	20.0	-	[16]
SiOC	Al_2_O_3_/Al	ZnO-SiO_2_-B_2_O_3_	Dipping	36.7	800/48	[25]
SiCN	YSZ/ZrSi_2_	-	Dipping	50	-	[28]
SiCN	YSZ/AYZ (Al2O3-Y2O3-ZrO2)	Glass G8470, Glass G018-311	Dipping	93	950/6	[23,24]

## Data Availability

The data presented in this study are available on request from the corresponding author.
